# Long noncoding RNA PVT1: potential oncogene in the development of acute lymphoblastic leukemia

**DOI:** 10.3906/biy-1801-46

**Published:** 2018-10-25

**Authors:** Narjes YAZDI, Mohammad HOUSHMAND, Amir ATASHI, Alireza KAZEMI, Ali Anjam NAJMEDINI, Mahin Nikougoftar ZARIF

**Affiliations:** 1 Blood Transfusion Research Center, High Institute for Research and Education in Transfusion Medicine , Tehran , Iran; 2 Department of Hematology and Blood Banking, School of Allied Medical Sciences, Shahid Beheshti University of Medical Sciences , Tehran , Iran; 3 Department of Molecular Genetics, Tehran Medical Branch, Islamic Azad University , Tehran , Iran; 4 Stem Cell and Tissue Engineering Research Center, Shahroud University of Medical Sciences , Shahroud , Iran

**Keywords:** Long noncoding RNA, PVT1, acute lymphoblastic leukemia, c-Myc, siRNA

## Abstract

Emerging evidence shows that long noncoding RNAs (lncRNAs) participate in various cellular processes, and that plasmacytoma variant translocation 1 (PVT1), a newly described oncogene that interacts with various molecules such as p15, p16, NOP2, and c-Myc, is a major contributing factor in tumor development. However, the role of this oncogene remains unknown in the pathogenesis of acute lymphoblastic leukemia (ALL), the most prevalent form of childhood leukemia. In this study, we first measure the expression level of PVT1 in a Jurkat cell line, then small interfering (siRNA) PVT1 is applied to demonstrate the impact of PVT1 knockdown in apoptosis, proliferation, the cell cycle, and its downstream targets. Our findings show that lncRNA was significantly higher in the ALL cell line than normal lymphocytes and that PVT1 knock-down increased the rate of apoptosis, caused G0/G1 arrest in the cell cycle, reduced the proliferation rate, and, above all, reduced the stability of c-Myc protein. All findings were confirmed at the molecular level. Our results may indicate the role of PVT1 knock-down in the suppression of ALL development and might provide an option for targeted therapy for leukemic conditions.

## 1. Introduction

Acute lymphoblastic leukemia (ALL), which occurs in both children and adults, is characterized by uncontrolled proliferation of T or B lymphoblasts. The incidence rate of this form of leukemia is much higher in children between 2 and 5 years of age and it is considered to be the most common cause of cancer deaths in children in the United States (Pui et al., 2008) . Wide genomic alterations such as somatic mutation in PAX5, deletion of E2A and IKZF1, and chromosomal rearrangements are considered hallmarks of ALL that perturb the diverse signaling pathways involved in vital cellular processes (Mullighan et al., 2007; Gu et al., 2016) . Various oncogenes, such as TAL1, LMO2, HOX A, and c-Myc, participate in the development of ALL. However, c-Myc, which is downstream of the Notch-1 signaling pathway, plays an important role in promoting cell growth and in the proliferation of malignant cells (Kamdje and Krampera, 2011; Gu et al., 2016) . Different studies showed that while this axis is augmented in about 50% of ALL cases, applying different c-Myc inhibitors increases cell death and is an effective therapeutic option for ALL patients (Delgado and León, 2010; Roderick et al., 2014) .

Long noncoding RNAs (lncRNAs) are noncoding transcripts larger than 200 nucleotides that have a role in a variety of biological processes such as the cell cycle, apoptosis, epigenetic regulation, and imprinting (Kung et al., 2013; Garzon et al., 2014) . Mounting evidence demonstrates the participation of various lncRNAs, including HOTAIR, H19, GAS5, and RUNXOR, in the pathogenesis of several malignancies such as breast cancer and leukemia (Wei and Wang, 2015) . Plasmacytoma variant translocation 1 (PVT1), located in the chromosomal region of 8q24 downstream of MYC, has various roles in both normal and malignant conditions (Zeng et al., 2015) . This cancer-related region has drawn the attention of researchers because of its role in DNA rearrangement, direct interaction with c-Myc, and production of about twenty lncRNAs and six microRNAs (Colombo et al., 2015) . It has been shown that the expression of lncRNA PVT1 is associated with enhanced proliferation and invasion of osteosarcoma, small cell lung cancer, and melanoma. Treatment with siRNA-PVT1 results in cell cycle arrest, apoptosis, and the suppression of proliferation (Huang et al., 2016; Zhou et al., 2016; Wang et al., 2018) . It has been elucidated that serum levels of PVT1 are increased in gastric cancer, small cell lung cancer, and cervical cancer, all of which are accompanied by low overall survival rates. ehTrefore, lncRNA PVT1 can be considered a diagnostic marker and a suitable therapeutic target (Kong et al., 2015; Cui et al., 2016; Yang et al., 2016) .

Due to the importance of c-Myc in ALL pathogenesis and considering the fact that lncRNA PVT1 potentiates and stabilizes this oncogene, we resolved to demonstrate for the first time the role of PVT1 knock-down in the suppression of ALL development.

## 2. Materials and methods

### 2.1. Cell culture

Jurkat cells were cultivated in a T25 flask in Roswell Park Memorial Institute (RPMI) 1640 medium with 10% fetal bovine serum (FBS) and 1% penicillin/streptomycin and maintained in a humidified incubator containing 5% CO 2 at 37 °C.

### 2.2. RNA interference

To determine the effect of PVT1 knock-down, we purchased two siRNAs against lncRNA PVT1 that interact with two different parts of the PVT1 mRNA sequence (Hs_PVT1_5 FlexiTube siRNA and Hs_PVT1_6 FlexiTube siRNA) (QIAGEN, Germany). AllStars negative control siRNA (QIAGEN) was used as the negative control. Twenty-four hours prior to the treatment, 105 cells were seeded in each well of a 24-well plate. Transfection of the siRNAs was performed by applying Lipofectamine 3000 according to the manufacturer’s protocol (Invitrogen, USA). All experiments were performed 48 h after siRNA transfection.

### 2.3. Real-time polymerase chain reaction

Total RNA from the transfected cells was extracted using QIAzol reagent (QIAGEN). After the qualification using a NanoDrop spectrophotometer (Thermo Fisher Scientific, USA), the extracted RNA was reverse-transcribed into cDNA using a cDNA synthesis kit (Takara, USA). Realtime polymerase chain reaction (RT-PCR) was performed by applying SYBR Green Master Mix (Takara). The results were normalized with beta actin (ACTB) as an internal control and the relative expression fold changes of the mRNAs and lncRNA were calculated using the 2–ΔΔCt method. The following primers were designed: PVT1: 5¢ GTGCTCTGTGTTCACCTGGTTCATC 3¢ (forward) and 5¢ GCCCGTTATTCTGTCCTTCTCATG 3¢ (reverse). c-Myc: 5¢ AGCGACTCTGAGGAGGAAC 3¢ (forward) and 5¢ CTGCGTAGTTGTGCTGATG 3¢ (reverse). Bcl2: 5¢ CAACATCACAGAGGAAGTAG 3¢ (forward) and 5¢ GGAACACTTGATTCTGGTG3¢ (reverse). Caspase-3: 5¢ ATTGATGCGTGATGTTTCTAAAG 3¢ (forward) and 5¢ CAATGCCACAGTCCAGTTC 3¢ (reverse). p15: 5¢ CTGGACCTGGTGGCT ACG 3¢ (forward) and 5¢ ACATTGGAGTGAACGCATCG3¢ (reverse). p16: 5¢ AAGGTC CCTCAGACATCC3¢ (forward) and 5¢ TCGGTGACTGATGATCTAAG3¢ (reverse).

### 2.4. c-Myc protein expression assay

Intracellular c-Myc protein expression was detected in the treated and control groups by flow cytometry using a primary antibody against human c-Myc protein and fluorescein isothiocyanate (FITC)-conjugated secondary antibody (Dako, Denmark). Before the antibody reaction, cells were treated with reagents from a permeabilization kit (Dako) according to the manufacturer’s instructions. The mean fluorescence intensity (MFI) of the antibody-reacted cells as a protein expression level and the percentage of reacted cells were evaluated in both the control and treated groups.

### 2.5. Western blotting

The total proteins were extracted from each study group using lysis buffer, and after quantification of the protein concentration using a bicinchoninic acid (BCA) protein assay kit (Thermo Scientific, USA), equivalent amounts of protein were subjected to 10% sodium dodecyl sulfate-polyacrylamide gel electrophoresis for 90 min. Subsequently, the separated proteins were transferred to a Millipore 0.45 µm polyvinylidene difluoride (PVDF) membrane and blocked in washing buffer containing 5% nonfat dry milk for 1 h at room temperature. The membranes were then confronted with anti-β-actin and anti-c-Myc (all from Abcam, USA) at 4 °C for 24 h and, following that, HRP-conjugated antimouse antibody at 25 °C for 2 h. Finally, the protein bands were visualized with Bio-Rad (USA) Trans-Blot and band intensities were quantified using TotalLAB software version 1.10 (TotalLAB, UK).

### 2.6. Apoptosis assay

An apoptosis assay was performed to analyze the role of PVT1 knock-down in the enhancement of apoptosis in leukemic cells. Briefly, the cells were washed using PBS and resuspended in the binding buffer. Then 5 µL of FITC-conjugated annexin V and 5 µL of propidium iodide were added to the samples and incubated for 10 min at room temperature. The experiment was carried out using flow cytometry.

### 2.7. Carboxyuflorescein diacetate N-succinimidyl ester cell division assay

To assess the role of PVT1 in the proliferation of Jurkat cells, a carboxyuflorescein diacetate N-succinimidyl ester (CFSE) cell division assay was performed after siRNA treatment according to a related protocol (Houshmand et al., 2017) . Briefly, samples were washed using PBS, CFSE 100 50 was added to the cell suspension as a cell division tracking reagent, and the mixture was incubated at 37 °C for 20 min. Subsequently, FBS was utilized in order to negate any extra CFSE in the solution. The cells were then centrifuged, the supernatant was removed, and the cells were added to each well of a 24-well plate.

### 2.8. Cell cycle analysis

To determine the effect of PVT1 knock-down in regulating the cell cycle and verify the results of the CFSE assay, a cell cycle analysis was performed. Briefly, samples were washed and resuspended in Tris buffer. Afterward, an RNAseand DNA-specific fluorochrome solution, composed of propidium iodide in 0.1% sodium citrate, plus 0.1% Triton X-100, was added to the cells. The samples were then incubated at 37 °C for 15 min and analyzed by flow cytometry.

### 2.9. Statistical analysis

Statistical analysis of the results was performed by an analysis of variance (ANOVA) test using SPSS software (IBM Corp., USA). Data are presented as mean ± standard deviation. P < 0.05 was considered significant. All graphs were designed using GraphPad Prism 7 software (GraphPad Inc., USA).

## 3. Results

### 3.1. Upregulation of PVT1 and c-Myc

**Figure 1:**
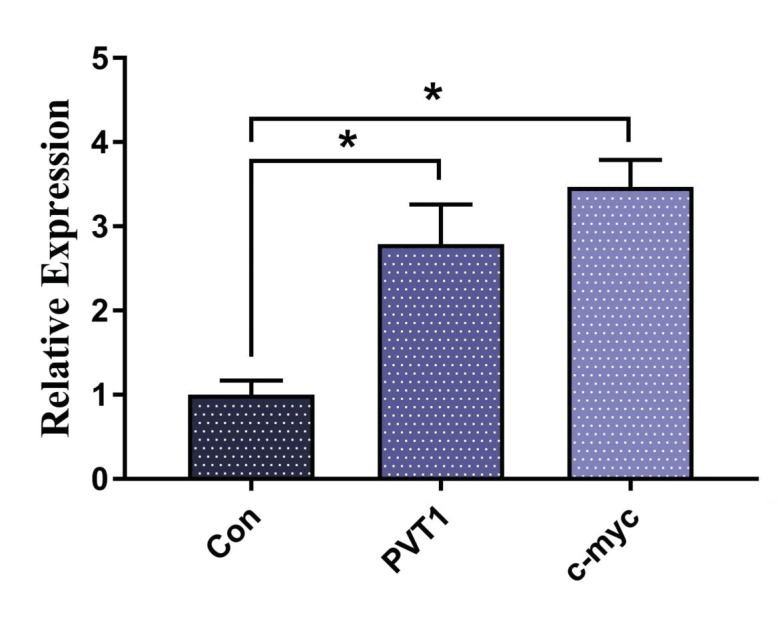
Relative expression of PVT1 and c-Myc in Jurkat cells. *: P < 0.05.

**Figure 2:**
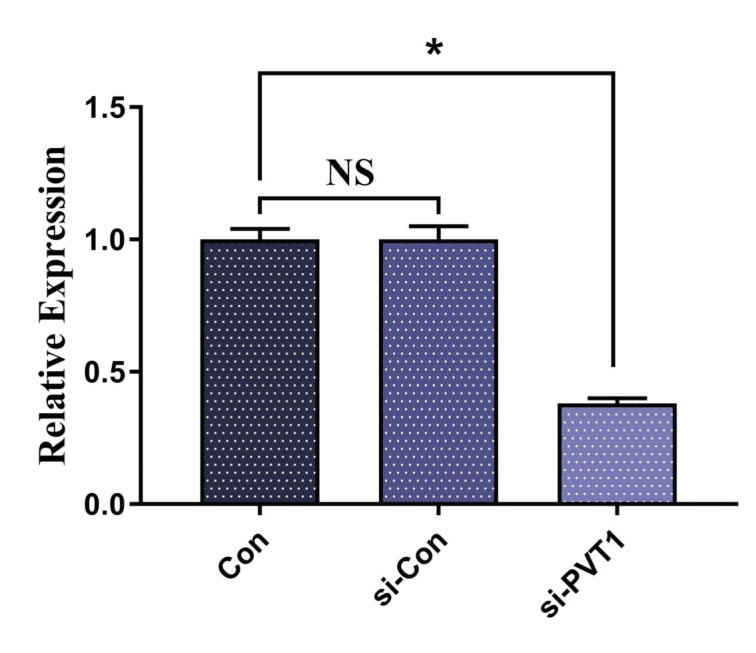
Relative expression of PVT1 in Jurkat cells after treatment with siRNA PVT1. *: P < 0.05.

The expression levels of lncRNA PVT1 and c-Myc in the Jurkat cell line were measured before knock-down of the lncRNA. The results demonstrate higher expression of PVT1 lncRNA in the ALL cell line than in normal cells. Meanwhile, the level of c-Myc was considerably higher compared to the control group (Figure 1), showing that as far as ALL is concerned, the c-Myc oncogene is most important. To verify the interaction of these two oncogenes, PVT1 knock-down was performed. The decrease in PVT1 expression is shown in Figure 2.

### 3.2. Knock-down of lncRNA PVT1 regulates c-Myc apoptotic and cell cycle genes

**Figure 3:**
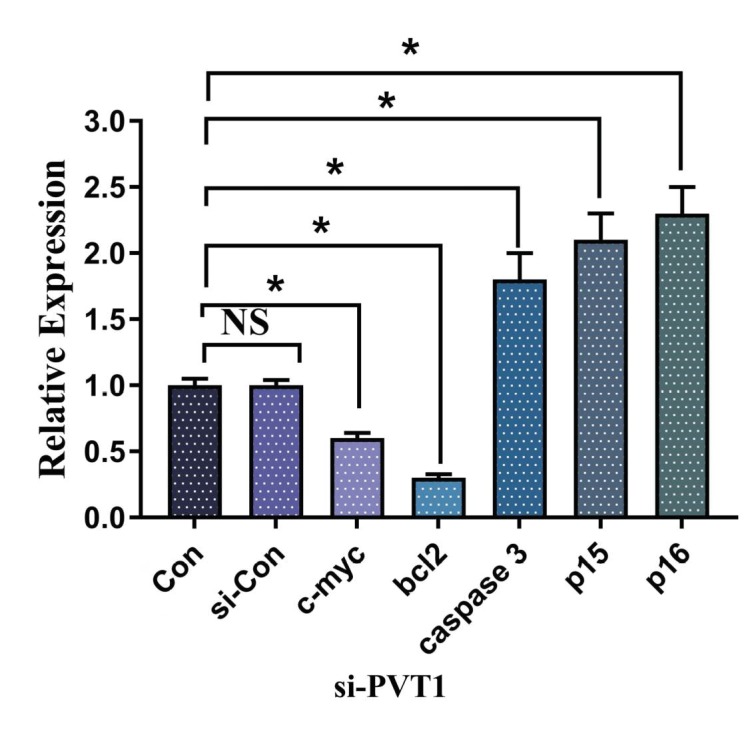
Relative expression of c-Myc, apoptotic, and cell cycle genes in Jurkat cells during siRNA PVT1 treatment. *: P < 0.05.

Our results demonstrate that PVT1 has a role in ALL pathogenesis, in which knock-down of lncRNA leads to the reduction of c-Myc and Bcl2, and augments caspase-3, p15, and p16 expression (Figure 3). As the c-Myc diminished significantly, it affected the development of cells, which was verified by the proliferation assay. Meanwhile, our experiment shows that PVT1 knockdown results in apoptosis initiation and changes in the cell cycle regulator genes; consequently, an apoptosis assay and cell cycle analysis were performed.

### 3.3. Downregulation of the c-Myc protein in PVT1 knock-down cells

**Figure 4:**
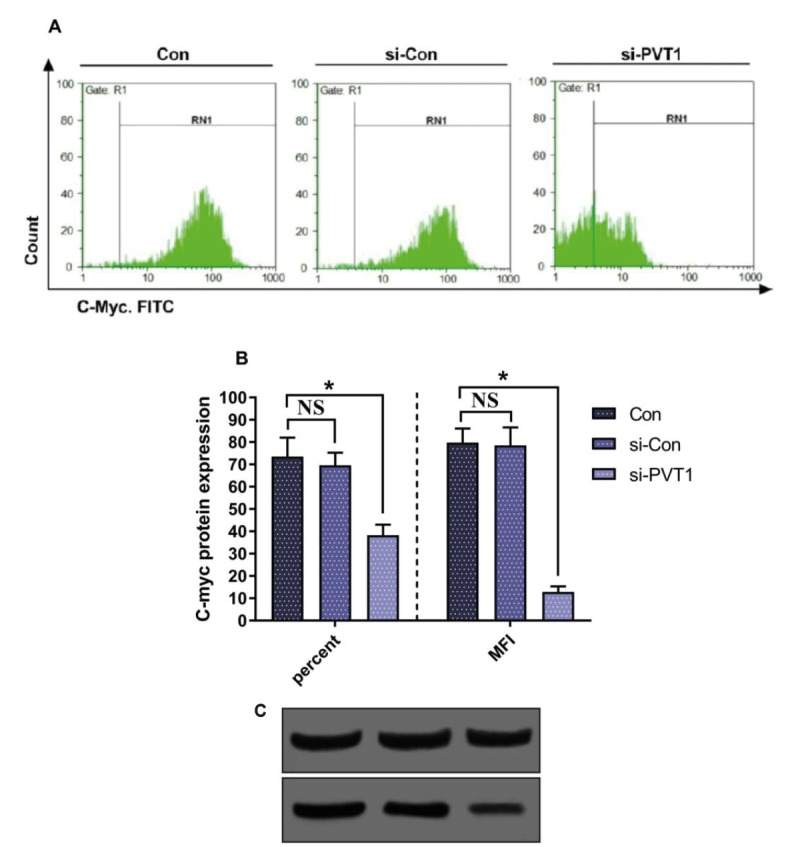
Expression of intracellular c-Myc protein within Jurkat cells treated with siRNA PVT1. A) Flow cytometry histograms; RN1 represents the percentage of c-Myc-positive cells. B) The percentage and MFI of positive cells in different groups. *: P < 0.05. C) Western blot for c-Myc expression in Jurkat cell line following standard protocol. Left band represents pretreatment, middle band shows cells treated with anti-PVT1-siRNA, and right band shows cells treated with PVT1-siRNA.

Flow cytometric detection of intracellular c-Myc revealed a decrease in the c-Myc-positive cell population within the siRNA PVT1-treated cells. In parallel, the MFI, which represents the median fluorescent intensity of the antibody-reacted cells, showed a significant decrease in siRNA PVT1-treated cells (Figure 4). Subsequently, we examined the protein expression level of c-Myc using western blot analysis. The results demonstrated that PVT1 knock-down significantly diminished c-Myc protein expression in the Jurkat cells.

### 3.4. PVT1 plays a role in apoptosis

**Figure 5:**
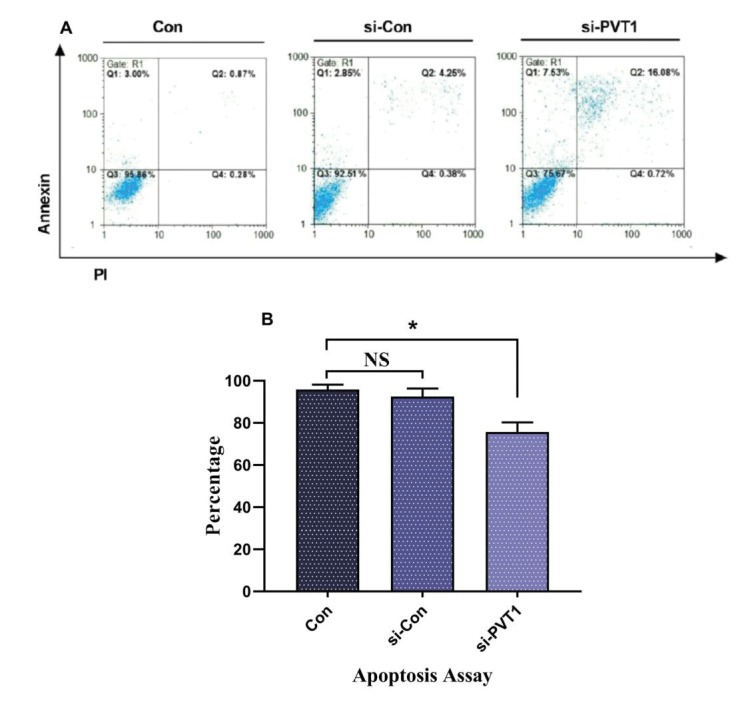
Apoptosis assay in Jurkat cells treated with PVT1 siRNA. A) Flow cytometry graphs; Q3 shows the percentage of viable cells, while proapoptotic, apoptotic, and necrotic populations are represented by Q1, Q2, and Q4, respectively. B) The summarized percentage of viable cells in different groups. *: P < 0.05.

To investigate the role of PVT1 knock-down and its effect on the fate of cells, an apoptosis assay was performed 48 h after siRNA treatment. The flow cytometry results verified the qRT-PCR and showed a marked increase in apoptosis in the Jurkat-transfected cells compared to the control group (Figure 5).

### 3.5. PVT1 knock-down effects proliferation

**Figure 6:**
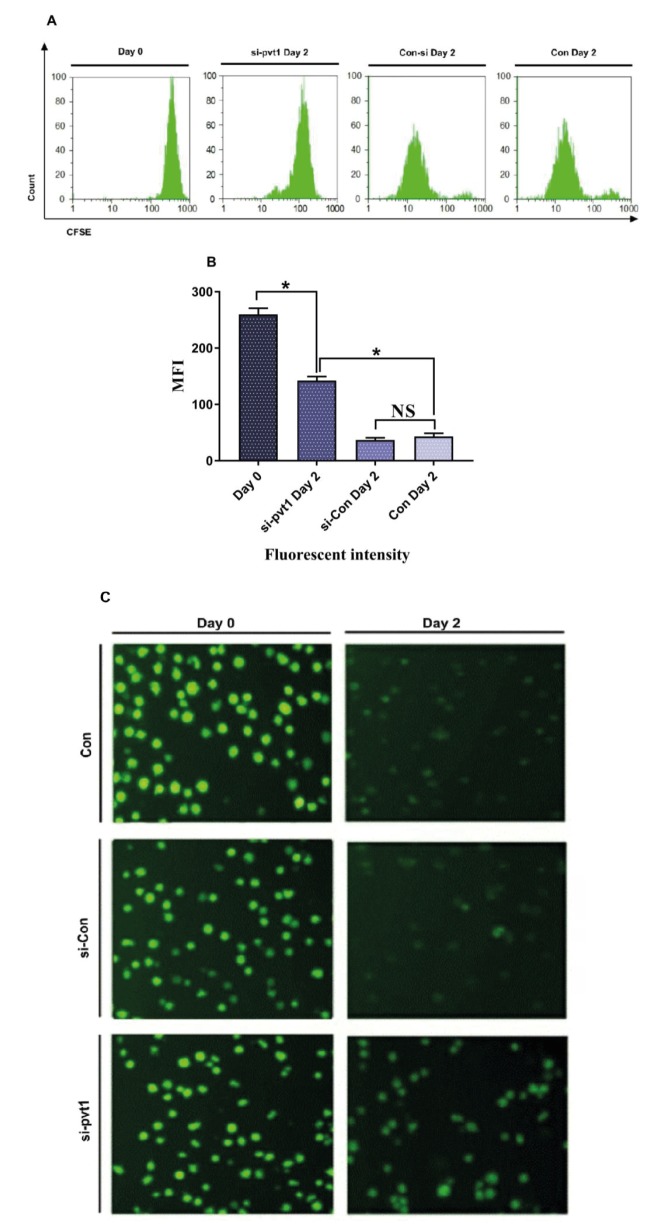
Proliferation rate in Jurkat cells treated with PVT1 siRNA by CFSE assay. A) Flow cytometry analysis of TF-1 cells on day 0 and after 48 h of treatment. B) The overall MFI of CFSE in different groups, showing the lower proliferation rate in PVT1 siRNA-treated cells. C) Fluorescent microscopy pictures of CFSE assay in different groups (20×).

To demonstrate the role of PVT1 in the enhancement of proliferation via diverse pathways, a CFSE assay was performed using flow cytometry and fluorescent microscopy after siRNA treatment. CFSE is a fluorescent dye that divides equally between cells during cell division. Therefore, reduction of fluorescent intensity is associated with the rate of proliferation. Our results show that fluorescent intensity was remarkably higher in the PVT1 knock-down cells compared to the control group. Assessment of CFSE via fluorescent microscopy confirmed the flow cytometry analysis and showed lower fluorescent intensity in the control group (Figure 6). This result indicates the impact of PVT1 knock-down in the suppression of proliferation and regulation of cell development.

### 3.6. Cell cycle regulation via lncRNA PVT1

**Figure 7:**
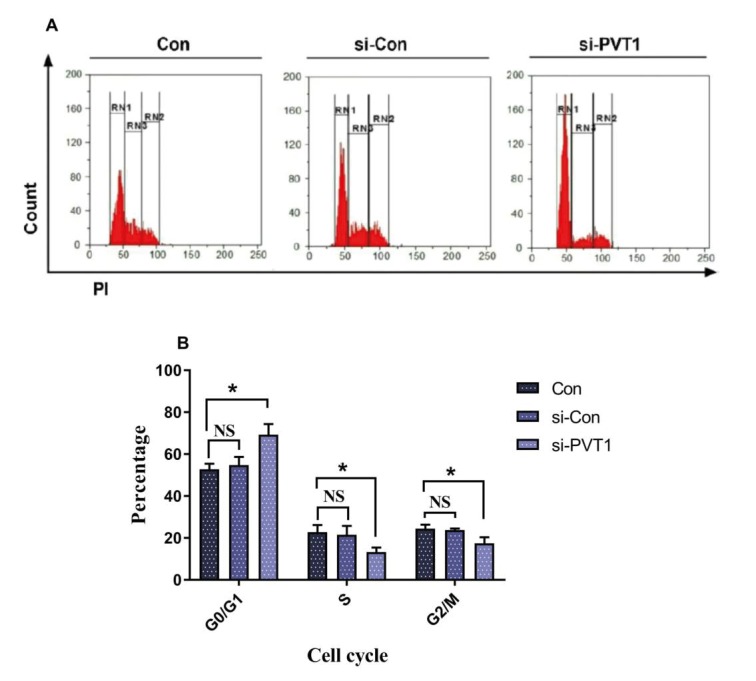
Cell cycle analysis of Jurkat cells in different conditions. A) RN1, RN2, and RN3 respectively represent G0/G1, G2/M, and S phases. B) Distribution of cells between cell cycle phases in groups. *: P < 0.05.

To confirm the results of the CFSE assay and assert the role of PVT1 in cell cycle regulation, a cell cycle analysis was performed. Our findings show that after PVT1 knockdown, there was a significant increase in the G0/G1 phase, and the S phase population diminished dramatically during the experiment compared to the control group (Figure 7). These results, like those of the CFSE assay, demonstrate the role of PVT1 in cell cycle regulation.

## 4. Discussion

ALL is a heterogeneous hematologic malignancy, recognized by impaired cell differentiation and proliferation processes, which engender the accumulation of cancerous cells in bone marrow and peripheral blood (Pui, 2000) . Although the potential for achieving complete remission (CR) in pediatric ALL cases is promising, because of relapse, CR declines to about 40% in adults, which is considered a major hurdle for successful treatment (Jabbour et al., 2015) . The conventional treatment protocol comprises the use of intensive chemotherapy agents with associated complications. Thus, emerging new therapies, such as monoclonal antibodies and molecular therapies, may promote more effective clinical outcomes (Pui and Jeha, 2007; Tatar et al., 2016) . Different lncRNAs take part in normal lymphoid cell development, but various experiments have shed light on some lncRNAs like LUNAR1 and NALT that, by interacting with NOTCH1, lend a hand in leukemia development (Wallaert et al., 2017) . In this study, for the first time, we show the interplay between lncRNA PVT1 and its downstream target in an ALL cell line and elucidate the role of PVT1 knock-down in the suppression of leukemic cell development. The 8q24 chromosomal region, as the location of PVT1 and c-Myc, includes a gene desert region and is a common site for different genetic aberrations (Huppi et al., 2012) . Various studies demonstrated the coexpression pattern of these two oncogenes, which may participate in the same signaling pathways (Riquelme et al., 2014; Colombo et al., 2015) . Our results show that the expression level of lncRNA PVT1 was significantly higher in the ALL cell line in comparison to the control group and in accordance with the results of other studies (Li et al., 2015; Guo et al., 2017) . The expression of lncRNA PVT1 correlated with c-Myc expression. Meanwhile, after using siRNA PVT1, not only was the expression level of lncRNA PVT1 and c-Myc mRNA downregulated, but the protein level of the c-Myc onwcogene was also remarkably reduced. The presence of lncRNA PVT1 is an indispensable part of c-Myc expression, so the upregulation of lncRNA in various cancers preserves c-Myc phosphorylation and the subsequent degradation of this oncogene (Hamilton et al., 2015) , which our findings confirmed.

Our experiments emphasize the role of PVT1 in apoptosis in the ALL cell line, in which the knock-down of lncRNA is accompanied by cell death. Overexpression of lncRNA PVT1 in ovarian cancer shows the role of this oncogene in apoptosis inhibition. In this regard, knock-down of lncRNA initiates the apoptosis process in colorectal and ovarian cancers (Takahashi et al., 2014; Liu et al., 2015) . Meanwhile, qRT-PCR confirmed the flow cytometry data that indicated significant enhancement of caspase-3 and reduction in Bcl2 expression levels. The mechanism of apoptosis initiation is attributed to the elevation of TGFβ1 as well as the degradation of the c-Myc protein within the PVT1 knock-down cells.

Accumulated evidence indicates that lncRNA also participates in the cell cycle and proliferation regulation in various cells (Colombo et al., 2015) . Different studies show that knock-down of PVT1 results in G0/G1 phase arrest, attenuating the progression of the cell cycle and, in turn, impairing proliferation ability (Zeng et al., 2015; Chen et al., 2017) . The CFSE assay showed that although fluorescent intensity decreased in all groups in comparison to day 0, the CFSE dye was highly preserved in the PVT1 knock-down cells in contrast to the control group, which highlights the role of PVT1 knock-down in proliferation inhibition. Meanwhile, the cell cycle analysis showed a reduction in the S and G2/M phases and G0/G1 arrest in PVT1 knock-down cells, which confirms the results of the proliferation analysis. As different studies have reported a link between PVT1 and various molecules, such as p21, p15, p16, and the NOP2 protein, in cell cycle regulation (Kong et al., 2015; Cui et al., 2016; Wu et al., 2017) , we measured the expression level of p15 and p16 in the ALL cell line. Our results show that the mRNA level of tumor suppressors p15 and p16 considerably increased in siPVT1 treated cells, which along with previous findings may show a part of a PVT1 target in regulating ALL cell development. On the other hand, c-Myc apparently controls cell proliferation and the cell cycle, which in normal cells are tightly regulated by a different mechanism and are elevated in almost all malignant conditions (Zörnig and Evan, 1996; Schmidt, 1999) . Other studies show that inhibition of c-Myc induces apoptosis, lowers the proliferation rate, and enhances G0/G1 arrest in cancerous cells (Wang et al., 2008; Jeong et al., 2010) .

In conclusion, through its interaction with different intracellular pathways, PVT1 potentiates tumorigenic activity and is considered an important RNA in leukemic cells. Therefore, inhibition of this oncogene would help to eliminate these malignant cells and provide a useful option for targeted therapy for leukemia.
